# Canid Reproductive Biology: Norm and Unique Aspects in Strategies and Mechanisms

**DOI:** 10.3390/ani11030653

**Published:** 2021-03-01

**Authors:** Jennifer B. Nagashima, Nucharin Songsasen

**Affiliations:** Center for Species Survival, Smithsonian Conservation Biology Institute, 1500 Remount Rd., Front Royal, VA 22630, USA; songsasenn@si.edu

**Keywords:** Canidae, seasonality, estrus, assisted reproductive technologies

## Abstract

**Simple Summary:**

The family Canidae, composed of dog-like species such as wolves, foxes, and jackals, demonstrates a significant variety in reproductive biology. In general, female canids experience very long periods of ovarian inactivity during the year; however, there are diverse patterns with regard to seasonality between species, as well as within an individual species depending on geographic region or housing status. Understanding of these differences is critical to the development of assisted reproductive technologies for canid conservation efforts. This review summarizes the current knowledge of canid reproduction, including reproductive cyclicity, seasonal breeding, male sperm traits, and recent developments in assisted reproductive technologies for canids.

**Abstract:**

The reproductive physiology of canids is unique compared to other mammalian species. Specifically, the reproductive cycle of female canids is characterized by extended periods of proestrus and estrus followed by obligatory diestrus and protracted ovarian inactivity (anestrus). Although canid reproduction follows this general pattern, studies have shown variations in reproductive biology among species and geographic regions. Understanding of these differences is critical to the development of assisted reproductive technologies including estrus induction, gamete rescue, and embryo production techniques for canid conservation efforts. This review summarizes current knowledge of canid reproduction, including estrus cyclicity, seasonality, and seminal traits, with the emphasis on species diversity. The application of reproductive technologies in wild canid conservation will also be discussed.

## 1. Introduction

**Figure 1:**
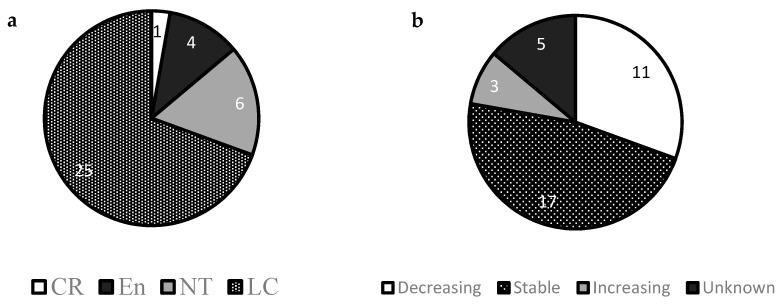
(**a**) Numbers of wild canid species listed as critically endangered (CR), endangered (En), near threatened (NT) or least concern (LC) status according to the IUCN red list of endangered species; (**b**) Numbers of canid species with decreasing, stable, increasing or unknown population trends.

Including the domestic dog, the canid family consists of 37 extant species, five of which are listed as “endangered” or “critically endangered” by the International Union of Conservation of Nature (IUCN) (Figure 1a). Furthermore, 1/3 of wild canid species are in decline due to increased anthropogenic activities, such as agricultural development, whereas two species, including the coyote (*Canis latrans*) and golden jackal (*Canis aureus*) are abundant with increasing population trends (Figure 1b).

Reproductive sciences play critical roles in species conservation and management [1,2]. Specifically, advances in the understanding about species’ reproductive biology may permit the development of reproductive technologies that are useful for ensuring genetic and demographic viability of ex situ wildlife populations [1]. Such knowledge can also assist in the development of strategies to control overpopulated wildlife species [1]. Yet, reproductive biology has not been studied in more than 90% of mammals on the planet, and the knowledge gained from “well-studied” species have highlighted remarkable diversity in reproductive mechanisms, even among related species [3]. For example, within the Genus *Canis,* the domestic dog (*Canis familiaris*) displays non-seasonal reproduction, whereas their wild cousins, including grey wolf (*Canis lupus*), red wolf (*Canis rufus)*, and coyote are seasonal breeders, and dingo [4] (*Canis lupus dingo)* exhibits variable reproductive seasonality. Though the domestic dog serves as a critical baseline for the reproductive physiology of the taxon as a whole, investigations on species-specific differences are critical for the development of reproductive technologies for managing endangered canid populations. Comparison of reproductive seasonality, puberty, and parental behaviors across Canidae have previously been reviewed [5]. However, there are also several aspects of domestic dog reproduction that as-yet elude understanding, including the mechanisms of estrus resumption and egg maturation prior to fertilization. In this review, we summarize current knowledge of canid reproductive biology, with the emphasis on species diversity, and the application of reproductive technologies in wild canid conservation.

## 2. Canid Reproductive Biology

The current knowledge about reproductive biology of the canid family is mostly gleaned from the studies in the domestic dog [6,7,8]. Generally, female canids are monestrus with the reproductive cycle characterized by an extended period of proestrus and then estrus (~1 week each). The estrous period is characterized by an estrogen peak that coincides with rising circulating progesterone concentration, prior to ovulation [6,8,9,10,11,12,13]. Estrus is followed by diestrus, a luteal phase averaging 2 months in duration irrespective of pregnancy [6,14]. Diestrus is succeeded by anestrus, an extended (2–10 months) interval of ovarian quiescence [6]. The reproductive cycle of most wild canids is consistent to that of the domestic dog where diestrus is followed by an extended period of ovarian inactivity. However, peripubertal bush dogs (*Speothos venaticus*) housed in zoological institutions can undergo multiple estrous cycles without the anestrous period [15]. Seasonal polyestrous cycles have also been shown in captive dholes (*Cuon alpinus*) and New Guinea singing dog (*Canis dingo hallstromi*) where females enter estrus when the prior breeding attempt does not result in pregnancy [16,17,18]. While female domestic dogs and most wild canids ovulate spontaneously in the absence of a male, there is evidence that the Island fox (*Urocyon littoralis*) [19] and maned wolf (*Chrysocyon brachyurus*) [20,21] are induced ovulators and require the presence of a male for estrus and/or ovulation to occur. To date, the mechanisms of induced ovulation in these two canids have not been elucidated. However, there has been evidence suggesting that an olfactory mechanism involving volatile chemicals excreted in the urine may play roles in this phenomenon. Specifically, female maned wolves that had visual access to a male as well as the ability to contact his urine scent marks deposited on the shared fence line ovulated, but other individuals housed at the same facility with only visual contact to males failed to ovulate [20]. Analysis of volatile organic compounds excreted in maned wolf urines revealed differences in between males and females, and several compounds that were more abundant in the males have been reported as semiochemicals in other mammals [22].

Like other mammals, the canid’s ovarian cycle is regulated by the hypothalamic–pituitary–gonadal axis [6,23,24,25]. Prior to proestrus, there is an increase in gonadotropin releasing hormone pulses from the hypothalamus which, in turn, stimulate follicle stimulating hormone and luteinizing hormone (LH) release from the anterior pituitary [6]. The increase in pituitary hormone pulses initiates follicular growth that stimulates gonadal steroidogenesis [26]. Continued rise in estradiol during proestrus triggers an LH surge that is followed by ovulation ~60 h later in dogs and 2 days in farmed Arctic foxes (*Vulpes lagopus*) [25].

### 2.1. Reproductive Seasonality

With the exception of the Basenji, whose annual estrus is controlled by decreasing day length [27], the domestic dog exhibits non-seasonal reproduction with males producing sperm year round while females enter estrus once or twice a year [6]. Unlike their domestic counterpart, most wild canids are seasonal breeders with the onset of a breeding season dependent on latitudinal location and/or variety of environment factors (e.g., rainfall and food availability; Table 1) [14]. The grey wolf becomes reproductively active in response to increasing day-length [14]; however, breeding date of this species shifts 22 days with each 10° increase in latitude [28]. Other North American canids, such as the red wolf [29], coyote [30], red fox (*Vulpes Vulpes* [31]) and island fox [19] also are reproductively active as day-length begins to increase. For South American canids, the time of breeding season varies among species. Specifically, the maned wolf breeds in the fall to early winter when day-length decreases [32]. The time of reproductive season in the crab-eating fox (*Cerdocyon thous*) varies depending on location [11,33]. Crab-eating foxes in Brazil breed once a year in winter and give birth in spring [11,33], whereas animals living in Argentina can reproduce twice a year when food is abundant [33]. On the opposite spectrum, the bush dog (*Speothos venaticus*) shows no reproductive seasonality and can breed year-round [34], and the New Guinea singing dog has a strict breeding season which does not shift with latitude [18]. Captive male wild dingos (*Canis familiaris dingo*) display no reproductive seasonality [35], whereas females experience distinct March–July breeding season in Central Australia, likely in response to draught periods [36].

To date, factors controlling reproductive seasonality have not been fully eludicated. It has been suggested that the intervals of ovarian cycle of Basenji and wild canids are part of an endogenous circannual cycle that is influenced by photoperiod [23]. Evidence supporting this assertion is that translocation of canids (maned wolves, African wild dogs, red foxes) from the Southern to Northern hemisphere, or vice versa, shifts the breeding season by 6 months [13,14,37]. The pineal gland and its hormone, melatonin, has been indicated as playing the central role in mediating neuroendocrine responses to changing daylight in seasonal breeders [38]. Melatonin-induced advancement in reproductive season has been reported in silver foxes (*Vulpes vulpes*) [39], raccoon dogs (*Nyctereutes procyonoides*) [40], and male Arctic foxes [41], but not in female farmed Arctic foxes [41]. Furthermore, exposing domesticated silver (i.e., red fox) males to short day light from February (natural breeding season) to June (non-breeding season) can prolong spermatogenesis [42]. However, pinealectomy fails to alter reproductive seasonality in both male and female grey wolves, suggesting that other pathways or factors also play roles in regulating reproductive seasonality in wild canids [43].

The linkage between reproductive seasonality and rain fall/food availability has been shown in some wild canids. For example, the Ethiopian wolf (*Canis simensis*) exhibits a strong association between reproduction and rainy season [44,45]. In this species, females give birth at the end of rainy season and the young become independent at the end of the dry season when prey availability is high. For the African wild dog (*Lycaon pictus*), strong seasonal reproduction is observed in animals living at latitudes from 7 to 25° S (e.g., Bostswana and Zimbabwe); however, dogs living at latitudes <2° (e.g., Kenya) do not exhibit a clear pattern of reproductive seasonality [46]. In seasonal breeder populations, births normally occur during the cool, dry season with no association with the variations in total rainfall [46]. Yet, only female African wild dogs exhibit strict seasonality in reproductive activity while males can produce sperm year-round, albeit with poor quality during the non-breeding season [13]. Some small canid species, such as the fennec fox (*Vulpes zurda*), bush dog, and crab-eating fox may exhibit variations in the number of reproductive cycle per year between animals living ex situ and in situ. Specifically, under a controlled environment in captivity, females of these canid species can cycle twice per year compared to once per year for counterparts living in the wild [12,33].

Spermatogenesis occurs year-round in aseasonal canids. However, spermatogenesis only takes place during breeding season in canids that are strict seasonal breeders [14,47,48]. In canids that breed seasonally, there are also temporal variations in testosterone concentration with the hormone level beginning to rise prior to breeding season and the peak concentration coinciding with maximum sperm production [30,40,49,50,51].

### 2.2. Gametogenesis and Embryogenesis

The female gamete of canids exhibits several unique characteristics compared to that of other carnivore species. Canid oocytes contain a large amount of cytoplasmic lipids compared to other mammalian species [74]. Lipid yolk bodies first appear in the cytoplasm of the primary oocyte and the amount of these lipid bodies increases throughout the entire process of oogenesis, giving its dark appearance which is distinct from that of other mammalian species [75,76,77,78,79,80]. Analysis of total lipids extracted from dogs of various breeds revealed that intracellular lipids include saturated fats, triglyceride, cholesterol, phospholipids, and glycolipid, and that types and amounts of lipid fractions are consistent among individuals within and between different breeds [77]. However, the distribution of lipid bodies varies among oocytes recovered from different reproductive stages [78]. Specifically, oocytes recovered during the follicular phase displayed a diffuse pattern of lipid bodies while those retrieved during anestrus or luteal period exhibited peripheral or perinuclear distribution [78]. Analysis of chemical lipid composition revealed that phosphatidylcholines containing 34 carbons, especially phosphatidylcholine (34:1), are the most abundant phospholipids in the dog oocyte [81]. This phosphatidylcholine also has been found to be among the most abundant lipid ions in cat, human, sheep, and cattle oocytes, although variations in unique ions with specific m/z values corresponding to individual lipid species and ion abundances among species have been observed [81,82].

Unlike other mammalian species, canid oocytes ovulate at an immature stage and require up to 48–72 h to completing nuclear maturation within the oviduct [79,80,83,84]. However, nuclear maturation interval can be slightly varied among species. Specifically, indirect evidence indicate that silver fox oocytes may complete maturation within 24 h as high conception rates can be observed within 24 h after ovulation in this species [85]. In the domestic dog, the oocyte remains fertile 4–5 days after nuclear maturation (i.e., 6–7 days post ovulation) [86].

Like other mammalian species, canid spermatogenesis is controlled by the hypothalamic–pituitary–gonadal axis [87]. In the domestic dog, sperm can be recovered when the males reach sexual maturation at around 6–8 months of age [87,88], with optimal sperm production observed at 15–16 months old in beagles [87]. However, sperm production may not occur until the males approach 2 years old in grey wolves [14]. Sperm maturation occurs in the epididymis and the gametes acquire fertilizing ability when they reach the cauda region [89]. Studies in the domestic dog have shown that the entire process of spermatogenesis takes 62 days and sperm spend 15 days in epididymal transit [87]. Following ejaculation, dog spermatozoa can survive in the female reproductive tract for up to 7 days [86].

Although in vitro studies have indicated that dog sperm can penetrate immature oocytes [90], in vivo fertilization does not occur until 83 h post-ovulation (or ~35 h after the LH surge), even in the presence of spermatozoa [84]. Information generated from an in vitro study has indicated that dog metaphase II oocytes may require an additional 12 to 24 h to fully acquire developmental competence, or 5–6 days following the LH surge [91]. Fertilization occurs at the middle or distal part of the oviduct in foxes [83] and dogs, respectively [84]. Two-pronuclei zygotes can be observed 92 h after ovulation (~6 days after the LH surge) in the dog, and between 29 and 73 h post-mating in the raccoon dog [80]. The duration during which canid early stage embryos remain within the oviduct may vary among species [80,84,85,86,92]. Specifically, domestic dog embryos remain in the oviduct until the 16-cell to morula stage, and migrate into the uterus 10–12 days post-LH surge and develop to the blastocyst stage 12–13 days post LH-surge [93]. In the farmed Arctic fox, embryos remain in the oviduct for 6–8 days after mating and enter the uterus at the morula stage [85]. However, red fox and raccoon dog [80] embryos enter the uterus at the 8–16 cell stage, 4–6 days post mating [85].

## 3. Assisted Reproductive Technologies

When a genetically valuable individual dies before successful breeding, or in situations where physical or behavioral obstacles prevent the production of offspring, it is of critical importance to rescue and preserve the genetic potential of the individual for later re-introduction into the population. Assisted reproductive technologies include, but are not limited to, monitoring of and manipulation of estrus, gamete rescue and preservation, artificial insemination (AI) and embryo production technologies such as in vitro fertilization (IVF) and intracytoplasmic sperm injection, or even cloning (i.e., somatic cell nuclear transfer), and embryo transfer. Some of these technologies have been recently reviewed in the domestic dog [94]. Here, we focus our review on recent advancements in these technologies in wild canids.

### 3.1. Genome Rescue

As previously noted, our ability to collect mature sperm and eggs from the vast majority of canid species is hampered by their strict seasonality and unique oocyte maturation system. Sperm can be collected relatively easily in non-seasonal breeders, or during breeding seasons. This has been accomplished via epididymal sperm collection following castration, electroejaculation, urethral catheterization, or, for some more tractable species, trained manual collection. Epididymal sperm collection involves the isolation of the cauda epididymis and vas deferens and either retrograde flushing or cutting into the epididymis and incubation in warmed medium to allow motile sperm to swim out. Electroejaculation, which has been the gold standard for many wild canid species, is accomplished via electrical stimulation of the prostate. Urethral catheterization, while necessitating animal anesthesia like electroejaculation, it is more simple, involving the passing of a catheter into the urethra 30 s to 1 min to allow capillary action to pull reserve sperm into the catheter.

Method utilized has significant impact on both sperm quality and concentration. While not the focus of the current review, in the tractable domestic dog, sperm collected manually has been shown to maintain motility for longer than sperm collected via electroejaculation [95]. Manual collection is regularly applied in dog artificial insemination breeding efforts, and even epididymal sperm collection [96] has resulted in live births for the domestic dog following artificial insemination. Though AI success has yet to be reported, urethral catheterization has yielded high concentration and viability sperm in the domestic dog as well [97].

In wild canids, electroejaculation allows for the collection of good quality and volume of semen in a variety of species (Table 2). Notably, the fluids from the prostate may also help support the functionality of the collected sperm, though it does not appear to improve post-thaw sperm metrics in the domestic dog [98]. However, urine contamination in the sample is a significant issue in electroejaculation, and the procedure requires specialized equipment, expertise, and anesthesia. Conversely, urethral catheterization has been applied successfully to collect low-volume but high-concentration and motility sperm in red wolves [99], African wild dogs [100], and maned wolves [100], with minimal urine contamination and seminal fluid. Recently comparison of both techniques was conducted for red wolves, and determined that urethral catheterization was valuable as a means to evaluate male fertility, but did not allow for the collection of sufficient sperm for future assisted reproduction technologies [99]. Further, while digital manipulation is likely to produce the best quality sperm sample overall, this method requires training of the animals, and thus is not suitable for species or individuals that are part of reintroduction efforts. Manual sperm collection has been described in farmed foxes [101] as well as racoon dogs [102], crab-eating foxes [103], and maned wolves [104].

While significant variation in number and motility of sperm collected within the various techniques is apparent, this is partly a function of differences in collection time/season, animal age/health/dominance statuses, and technique (i.e., different stimulation protocols for electroejaculation). Nevertheless, no obvious patterns are evident between small, fox-like canid output and the larger bodied wolves for either of the highlighted metrics (Table 2). This may partly be due to the inverse relationship between body and testis size in mammals [105], which has previously been noted to explain the larger relative gonad weight of the crab-eating fox than that of the maned wolf [106].

Thus far, epididymal sperm collection has been described only for the domestic dog [117], farmed Arctic fox [118] (note: Ultrastructural study, did not report sperm concentration or motility), and grey wolf [115]. Unsurprisingly, while this technique allows for the rescue of motile sperm, it is limited in use by animal age (post pubertal only) and breeding season status (where applicable). Motile epididymal sperm have been collected from African wild dogs and maned wolves (Table 3, *unpublished data*). Briefly, testes were removed during necropsy or via castration, wrapped in saline-soaked gauze, and shipped overnight on ice. The following day, the vas deferens and cauda epididymis were isolated and motile sperm allowed to swim out into warmed (37 °C) phosphate buffered saline, then evaluated for concentration and motility. Interestingly, sperm presence is not always strictly correlated with season, as maned wolf sperm have been collected in June and August, outside the September to February, normal breeding season for the Northern hemisphere. Overall, sperm counts and motility are lower than electroejaculated samples from the same species (For comparison, refer to Table 2). However, the fact that the testes were collected primarily for management purposes (i.e., from surplus young or post-reproductive males), or following animal death (i.e., from old or sick animals) may also have contributed to the reduced values compared with electroejaculated sperm from healthy males in reproductive prime. While these epididymal concentrations would not be sufficient for artificial insemination via vaginal deposition, they are potentially enough for use in in vitro fertilization or, once developed in the future, oviductal AI.

On the female side, collection of immature oocytes can be done following spay or animal death, typically by mincing the outer surface of the ovary to release oocytesObtaining competent oocytes is significantly more challenging. In the Mexican grey wolf, aspiration of oocytes from post-spay ovaries during a natural breeding season or following estrus stimulation protocols have been performed [119]. To our knowledge, in situ oocyte aspiration has not been reported in canids, including the domestic dog, due to the additional challenge of the ovarian bursa, a fat pad encompassing the canid ovary and through which the oviduct runs. Oocytes can be collected surgically in dogs via laparotomy and retrograde flushing of the oviduct [120]. However, less-invasive, laparoscopic oocyte aspiration techniques utilized in other taxa (such as that which allowed for the collection of oocytes for the recent successful cheetah in vitro fertilization [121], will require significant additional fine-tuning to get through the ovarian bursa without damaging the future reproductive potential of the animal. Still, laparoscopic ovariectomies are becoming increasingly common in domestic veterinary practice [122], and continued advances in this area will support future applications to endangered canid assisted reproductive technology development.

**Figure 2:**
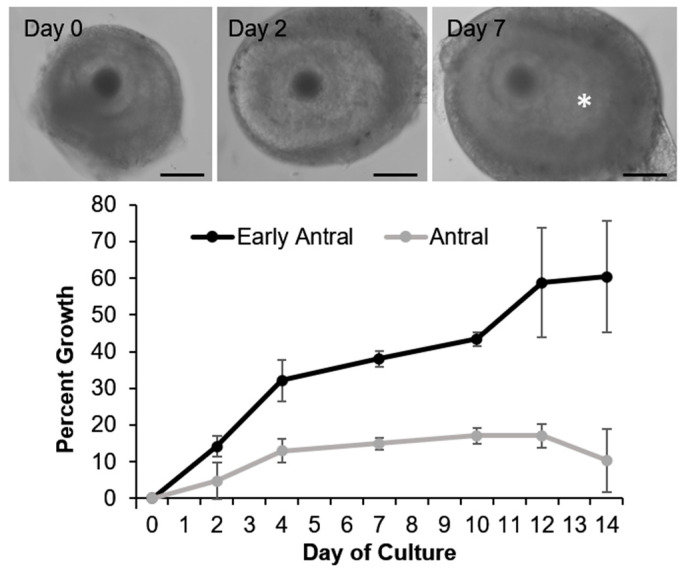
In vitro growth of isolated ovarian follicles (n = 2 early antral and 3 antral stage) from an African painted dog (SB#2516) over 14 days culture, with representative images of an early antral stage follicle developing to the small antral stage over 7 day culture. Asterisk (*) denotes antral cavity. Black bar = 100 µm. (*unpublished data*)

Owing to the challenge of obtaining competent gametes outside of breeding season (i.e., the majority of the year for most canid species), there is interest in growing sperm and oocytes in vitro. Ideally, even if the animal was pre-pubertal or not in breeding season, gonadal tissues can be incubated under appropriate hormonal and growth factor conditions to produce mature gametes. For example, murine neonatal testicular tissue has been cultured on an agarose gel block to produce mature sperm in vitro [123] which in turn produced live offspring. Currently, the majority of research in gonadal tissue culture has focused on the domestic dog [124,125,126,127,128,129,130,131] as a model/starting point for developing the technologies in endangered canid species. Using culture conditions developed for the dog [124], we have cultured ovarian follicles isolated from an African wild dog ovary (Figure 2, *unpublished data*). Specifically, small, growing ovarian follicles were mechanically isolated from surrounding ovarian tissue, then encapsulated in 3D hydrogel for structural support and incubated individually in minimum essential medium-based growth medium supplemented with 1 µg/mL porcine FSH and 100 ng/mL recombinant human Activin. Isolated early antral (diameter at isolation: >230 µm) and small antral stage (>500 µm) follicles increased in diameter and displayed development of antral cavities (a necessary development for the production of a maturation-competent oocyte, and denoted by * in Figure 2) over 14 days culture. Cavity development, as well as the increased responsiveness of early antral stage follicles over antral stages in the current culture system, is similar to what has been observed with domestic dog follicles [124].

Nevertheless, ovarian and testicular tissue culture has not yet advanced to a stage where mature, fertilizable gametes can be grown from large mammalian species. In the female, this is likely due to the prolonged incubation necessary to grow large mammalian follicles from the earliest (primordial) stage through to preovulatory, which is estimated to take about 100 days in species like the dog, cow, and human compared with ~20 days in the laboratory mouse [132]. Further, while mouse preovulatory follicles reach the final diameter of ~0.5 mm [132], the ~25 µm diameter dog primordial follicles [133] grow to an average preovulatory size of 3.5 mm [134]. This over 100× expansion in size comes with significant changes in physical, hormonal, and nutrient requirements which are challenging to recapitulate in vitro. As such, recent research has focused on the development of more “biomimetic” culture systems. One primary example is the implementation of bioreactor and microfluidic chip culture methods, wherein culture medium is designed to “flow” through the tissue culture chamber to better mimic nutrient availability and waste exchange rates that the tissue would be exposed to via circulation in vivo. On the female side, a prototype microfluidic ovary-on-a-chip has been developed for the in vitro culture of domestic dog ovarian tissue and isolated follicles [127]. Dynamic culture for ovarian tissues is ongoing in the dog and other large-mammalian models to fine-tune this and other novel culture methods to support in vitro folliculogenesis.

On the male side, this organ-on-a-chip technology has been used to support murine spermatogenesis in vitro for 6 months, including producing live offspring from sperm developed in the system [135]. There have been no published reports of similar technology being applied to domestic dog testicular tissue culture; however, grafting of canine testicular tissue into immunodeficient mice has been utilized to grow dog tissue under more “natural”/in vivo conditions [136]. In that study, progression of spermatogenesis was observed in tissues from immature but not adult donors, including presence of elongated spermatids after 4–8 months grafting.

### 3.2. Cryopreservation

There have been significant efforts to preserve canid sperm via cryopreservation. Techniques typically have either involved loading extended sperm with cryoprotectants into a straw prior to cooling and submersion in liquid nitrogen (so-called “closed” cryopreservation systems because cells do not come into direct contact with liquid nitrogen), or the pelleting of sperm in indentations in dry ice, followed by plunging into liquid nitrogen directly (“open” cryopreservation). There is an vast body of literature on sperm cryopreservation in the domestic dog, which have been previously reviewed [137]. More recently, research has focused on developing sperm cryopreservation protocols that do not utilize animal-based proteins, such as the gold-standard egg yolk which protects cells against cold-shock [138]. The rationale for developing egg yolk-free media is the risk of disease or microbial transmission and subsequent additional challenges with exportation regulations, as well as the biological variability inherent with the use of an animal-sourced reagent. Common egg yolk substitutes include soy lecithin [139,140,141] and polyvinyl alcohol [142], which have been comparable to yolk based extenders with regard to post-thaw survival, membrane protection and motility.

Thus far, all methods employed with wild canid sperm cryopreservation have utilized egg yolk in the extender solution, but cryoprotectant solutions have varied between species. For example, glycerol was superior to dimethyl sulfoxide (DMSO) in the cryopreservation of red wolf sperm [99], yet the opposite was true for the maned wolf [110]. In the domestic dog, DMSO is toxic [143], and therefore the majority of work has utilized glycerol as a starting-point for developing sperm cryopreservation in wild canids, including for the African wild dog [107,108,144], and grey and Mexican grey wolves [111]. Though application of artificial insemination in wild canids is yet limited, cryopreserved grey wolf [145] and farmed red fox sperm [146] have been successfully used to produce live offspring, highlighting the significant potential value to genetic management in endangered canid species.

Interestingly, though initial sperm survival following warming is fairly par-for-the-mammalian-course, both African wild dog [107,144] and red wolf studies [99,147] have noted rapid declines in sperm motility/viability in the first two hours of post-thaw incubation. This is not necessarily due entirely to damage resulting from the freezing process, as freshly collected red wolf sperm also experience a rapid loss in viability when maintained at near-physiological temperatures [147]. Recently, the influence of extracellular vesicles (EVs) from domestic dog oviducts on red wolf sperm post-thaw was evaluated. EVs are membrane-bound, nano- and micro-meter sized particles secreted from cells which carry protein, RNA, and DNA messages to neighboring cells. Understanding of the role(s) EVs play in modulating communication between gametes and embryos and the reproductive tract are only now being elucidated (see reviews [148,149]). The presence of domestic dog oviduct-derived EVs during thawing and incubation supported maintenance of red wolf sperm motility over ≥ 2 h incubation [150]. It was postulated that several proteins identified in the EVs with known sperm-motility and cell stress-modulating functions may have been responsible for the positive effects. While more investigations are necessary, these early results are promising for the development of an improved method to cryopreserve sperm in wild canids.

Comparatively less research has been done on wild canid oocyte preservation, although progress on the development of domestic dog ova cryopreservation has been reported [151,152]. This is in no small part due to the challenge of obtaining canid oocytes as well as the ability to in vitro mature the gamete, a step that is necessary for the evaluation of the cryopreservation success. As an additional complication, the lipid-richness of canid oocytes described previously has been suggested to increase oocyte sensitivity to chilling injuries [152]. Nevertheless, there has been one report of oocyte vitrification in the Mexican grey wolf wherein oocytes were equilibrated in a 7.5% ethylene glycol and DMSO solution prior to vitrification with a CryoTop (open) system in 15% ethylene glycol, 15% DMSO, and 0.5 M sucrose [119]. Oocyte viability post-thaw, as determined via propidium iodine staining, was similar to fresh controls. Unfortunately, owing to the lack of established in vitro maturation protocols in canids, the functionality of warmed oocytes has yet to be determined.

Domestic dog embryos (2–16 cell stages) vitrified in both open [153,154] and closed systems [91,155] have resulted in live births. Birth rates from these early stage embryos range from 6–36% based on embryo stage and location of transfer (i.e., uterine versus oviduct). This latter point is not trivial. Transfer of 2- to 16-cell stages to their physiologically-appropriate location (i.e., the oviduct, see section *Gametogenesis and Embryogenesis*) is technologically challenging, currently requiring laparotomy to access the oviduct through the dog’s bursal fat pad [156]. Standing/non-surgical transfer of embryos into the uterus has been successful in the domestic dog [153]; however, although transfer of fresh zygotes to 4-cell stage embryos to the uterine horns can produce pregnancy, the success rates are lower than those achieved from transferring advanced stage embryos (5.7 versus 23.5% for 4-cell to 8-cell embryos, per [157]). As such, there is interest in developing cryopreservation protocols for blastocysts which in turn would permit uterine transfer. Unfortunately, dog blastocysts have been shown to respond poorly to cryopreservation [153,155,158], typically displaying either abnormal morphology or reduced cell viability post-warming. In another study, dog blastocysts slow frozen with glycerol as a cryoprotectant remained intact post-thawing, whereas >80% exposed to ethylene glycol cryoprotectant ruptured [159]. To our knowledge, there have been no reports of live births from embryo cryopreservation in wild canid species; however, both an open vitrification system and programmable freezer method have been applied to Arctic fox embryos which resulted in implantation following embryo transfer [160]. Additional work is necessary to both improve blastocyst cryopreservation methods and determine the embryo cryopreservation technique(s) that will translate well to endangered canid assisted reproduction efforts.

As gametes and embryos are not always available and gonadal in vitro maturation techniques are not yet developed, testicular and ovarian tissue preservation holds high potential for genome preservation in canid species (See reviews [161,162,163]). Tissue cryopreservation/vitrification is complicated by the large mass, challenging consistency of CPA exposure and cooling/warming rates within the sample, as well as the heterozygous cell populations with potential differences in cryotolerance [164]. Nevertheless, the ability to successfully bank gonadal tissue in this way, combined with tissue transplantation or future in vitro culture technologies, would allow for the re-infusion of genetics long-term.

**Figure 3:**
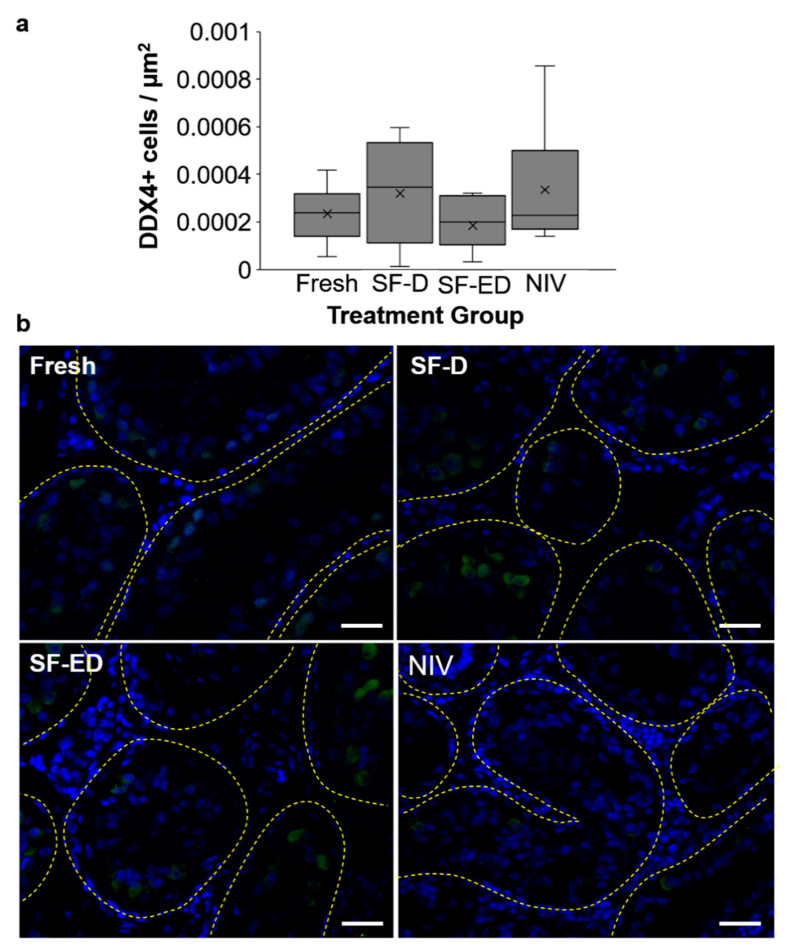
Grey wolf testicular tissue preservation, with (**a**) density germ cell marker DDX4 positive cells in grey wolf tissues following slow freezing in DMSO alone (SF-D), or in ethylene glycol and DMSO (SF-ED), or needle immersion vitrification (NIV) compared with fresh controls (n = 7 wolves), and (**b**) representative images of DDX4+ cells (green) with nuclear DAPI (blue) counterstain. Yellow line denotes outline of seminiferous tubules, and white bar = 50 µm. Reprinted with the permission from ref. [168]. Copyright 2021 Elsevier.

Again, most of the research in the area of gonadal tissue preservation for canids has been done in the domestic dog and taking advantage of xenografting to assess post-warming functionality of tissues. Ishijima et al. [165] evaluated the developmental potential of vitrified ovarian tissues after grafting into the ovarian bursa of immunodeficient mice and observed cell proliferation and morphologically normal ovarian follicles in grafts 4 weeks after transplantation. More recently, needle vitrification was compared directly with slow freezing [166]. The researchers found dog ovarian tissues vitrified in ethylene glycol, DMSO, and polyvinylpyrrolidone demonstrated follicle activation and progression to primary/secondary stages of development 9 weeks after grafting into immunodeficient rats [166]. For male domestic dogs, dissociated testicular cells have been vitrified in a commercial medium for stem cell culture (StemPro-34) then transplanted into chemically-castrated testes of immunodeficient mice after warming [167]. Transplanted cells formed seminiferous tubules containing donor germ cells 20 weeks after transplantation. There is one report of testicular tissue cryopreservation in grey wolves [168], wherein slow freezing (closed system) with 15% DMSO (SF-D), or a combination of 7.5% ethylene glycol and 7.5% DMSO (SF-ED), or needle immersion vitrification (open system) with 15% ethylene glycol, 15% DMSO, and 0.5M sucrose (NIV) all similarly maintained germ cell density compared with fresh (DDX4 marker, Figure 3). However, tissue architecture and levels of apoptosis post-thaw were improved in SF-ED compared with DMSO alone or NIV, indicating slow freezing with a combination of CPAs better supports viability of cryopreserved canid testicular tissues [168]. While significant work is yet needed to optimize these protocols for wild canids, ideally banked gonadal tissues can be either grafted into donors or grown in vitro to produce mature gametes in the future as a means of genetic rescue.

### 3.3. Estrus Induction

The ability to control canid estrus cycles would allow for the precise control of estrus and/or ovulation, which is necessary for optimal timing of artificial insemination or oocyte pickup. As with earlier discussed techniques, the majority of work in this area has been done in domestic dogs (reviewed in [169,170,171]). A variety of approaches to induce canine estrus have been reported, including administration of exogenous gonadotropins, dopamine agonists, and gonadotropin releasing hormone (GnRH) and its analogs. The latter approach works via acting on pituitary to release follicle stimulating hormone and luteinizing hormone, which promotes ovarian follicle development. Interestingly, prolonged exposure results in reproductive suppression and as such has been applied as a reversible contraceptive [172]. In short exposures (at least 7 days [173]) to GnRH agonists, domestic dogs in anestrus can display high rates of ovulation (up to 100% [174]). However, if treated during diestrus [174] or early anestrous [175], both ovulation rates and pregnancy success are significantly reduced compared with mid- or late-anestrus treatment.

The GnRH agonist deslorelin has also been used to induce estrus in several wild canid species during their natural breeding seasons. In grey wolves, estrus induction was achieved in at least 3/5 animals, and subsequently resulted in pregnancies [176]. In the coyotes, deslorelin implanted females display increased steroid hormone levels and have achieved successful pregnancies via artificial insemination with grey wolf sperm [177]. In the maned wolf, deslorelin implants stimulated ovarian activity, but ovulation only occurred in females paired with males or treated with an injection of luteinizing hormone to induce ovulation [20]. Deslorelin-treated Mexican grey wolves also had increased numbers of oocytes collected from their ovaries compared to those in natural cycles [119]. Importantly, administration of deslorelin to non-breeding season (i.e., mid-late anestrus, in October) coyotes resulted in transient elevations in steroid hormone concentrations, reflecting an ovarian response, and the display of sexual behaviors [178]. Such a technique could be valuable to obtain additional breeding opportunities in strictly seasonal species. While no pregnancies were achieved, copulatory ties were observed in two of the six coyote pairs. Though these data suggest additional, out-of-season breeding opportunities might be made possible in managed canids, treated females subsequently displayed suppressed reproductive behaviors during the following breeding season. In the study this behavior change did not significantly impact reproductive success, but it was noted that such shifts could have long-standing impacts on social hierarchy. In sum, while studies in the dog and wild canid demonstrate that estrus induction is possible, improved understanding of the physiology of anestrus is needed to improve on existing techniques. Further, the behavioral implications of out-of-season or significantly shifted estrous cycles must be considered in animal management and breeding efforts.

### 3.4. Artificial Insemination

While artificial insemination (AI) is commonly applied to domestic dog and fox breeding, there have been only a handful of successful reports of live offspring from AI in wild canids (see reviews [48,179]). For example, Asa et al. [176] reported the birth of grey wolf pups following estrus induction via deslorelin implant and deposition of 750 × 10^6^ sperm with high motility in the cauda vagina. As previously noted, this concentration may be feasibly obtained by electroejaculation in many canid species, but would not likely be possible utilizing sperm collected via urethral catheterization unless multiple collections were combined. Evaluation of frozen–thawed sperm concentrations necessary for intrauterine AI has previously been performed in farmed foxes [101]. The authors concluded that, taking into account sperm loss due to cryodamage and reduced fertilizing capacity, the minimum number of sperm necessary to achieve normal pregnancy rates and litter sizes is at least double what would be needed if using fresh sperm. This previous work in the dog and fox have laid the groundwork for successful implementation of the sperm-deposition technique, but the primary challenge in adapting AI for additional non-domesticated canid species is the need for intensive estrus monitoring and/or consistent estrus induction protocols. However, with increased sperm collection and banking efforts across Canidae, reports of additional success with this technique in other species is likely forthcoming.

### 3.5. In Vitro Oocyte Maturation and Fertilization

As noted earlier, the mechanism of canid oocyte delayed maturation in vivo is not yet fully understood, and as such it has been challenging to recapitulate the process in vitro. In vitro maturation involves the incubation of groups of oocytes in maturation media (typically TCM199) containing a protein source (e.g., bovine serum albumin), follicle stimulating hormone and luteinizing hormone or eCG/hCG, with incubation at 37–38 °C and 5% CO_2_ for 48–72 h. Rates of meiosis resumption and oocytes reaching Metaphase II varies depending on size of the source ovarian follicle [180] and animal age [181], but not reproductive stage of the donor [182]. Unfortunately, a protocol for producing consistently high rates of mature oocytes from the domestic dog has not been achieved. Still, embryos have been produced from in vitro matured oocytes in the domestic dog [183,184,185,186], and live offspring have been produced via IVF with in vivo matured oocytes [82,151]. In the latter, in vivo matured oocytes were collected from the oviducts of domestic dogs 6 days after the LH surge, and fertilized with 100,000 sperm that had been incubated under capacitation-stimulating conditions for ~3 h [82]. As yet, intracytoplasmic sperm injection has not produced offspring in the domestic dog.

Though obtaining in vivo matured oocytes in endangered canids is not yet technically feasible, and development of consistent in vitro maturation methods are still ongoing, translation of this technology to wild canid conservation efforts has been initiated in sperm capacitation studies. Recently, the in vitro survival and function of red wolf sperm in several different gamete handling medium was assessed [187]. It was found that the media previously utilized for domestic dog sperm capacitation and in vitro fertilization both supported red wolf sperm survival and early capacitation during extended incubation at physiological temperatures. This is promising for translating the success of IVF in the domestic dog to endangered canids, and also for improving in vitro incubation methods for wild canids in general, which would aid in the evaluation of handling and cryopreservation methods as they are being developed.

### 3.6. Cloning

Cloning, or somatic cell nuclear transfer, involves the transfer of somatic cell nuclei into enucleated oocytes and then activation to resume development. While there are some commercial entities using this technique to clone domestic dogs, efficiency of producing cloned embryos and live offspring via cloning is ~4% [153]. Grey wolves have been cloned using fibroblasts (collected and cultured from both donor ear cells and post-mortem abdominal skin) and enucleated, in vivo matured domestic dog oocytes [154,155]. Still, this technology overall is likely a long way from being applied to most endangered canid species’ conservation efforts. This technique would primarily be valuable in re-infusing genetics from founder individuals into the population, which is only feasible if properly preserved somatic cells exist from these animals and one can obtain mature canid oocytes. Some biobanks, like the Frozen Zoo at the San Diego Zoo institute for Conservation are poised to take advantage of somatic cell nuclear transfer and induced pluripotent stem cell technologies [156]. Further, efforts to improve in vitro maturation success in canid oocytes, which would support the advancement of many of the assisted reproductive technologies described here, are ongoing.

## 4. Conclusions

There is an incredibly variety of reproductive strategies within the Canidae family, from mechanisms of breeding season initiation to ovulation induction, despite the majority of members being under-studied. Though assisted reproductive technologies have advanced for the domestic dog and select wild canid species in recent years, understanding of each species’ unique traits will be necessary to implement techniques for population management in the future.

## Figures and Tables

**Table 1:** Geographic location and published seasonality in Canidae, organized by taxonomic clade based on [52,53,54].

Taxonomic Group	Species	Scientific Name	Geographical Location	Breeding Seasonality	Time of Breeding Season	Citation
Red fox-like canids	Arctic fox	*Vulpes lagopus*	Arctic Tundra	Yes	February–May	[55]
	Blanford’s fox	*Vulpes cana*	Middle East	Yes	January–February	[56]
	Corsac fox	*Vulpes corsac*	Central Asia	Yes	January–March	[57]
	Fennec fox	*Vulpes zerda*	Africa	Yes	January–July, September	[12]
	Kit fox	*Vulpes macrotis*	North America	Yes	December–January	[58]
	Red fox	*Vulpes vulpes*	Entire Northern hemisphere from Arctic Circle to Asiatic steppes and Australia	Yes	Late January–early March	[31,59]
	Ruppell’s fox	*Vulpes rueppellii*	North Africa and Middle East	Yes	December–February	[60]
	Swift fox	*Vulpes velox*	North America	Yes	December–March	[61]
	Tibetan sand fox	*Vulpes ferrilata*	Steppes and semideserts of the Tibetan plateau	Yes	Late February–March	[62]
Wolf-like canids	African wild dog	*Lycaon pictus*	Africa	Yes	Southern hemisphere, Feb–May; Northern hemisphere, late August–early October	[13]
	Black backed jackal	*Canis mesomelas*	Africa	Yes	June–July	[63]
	Coyote	*Canis latrans*	North America	Yes	December–April	[30]
	Dhole	*Cuon alpinus*	Central and Southeast Asia	Yes	Varies	[16,17,64]
	Dog, domestic	*Canis familiaris*	Global	No	-	[8]
	Dog, Dingo	*Canis lupus dingo*	Australia and Southeast Asia	Varies	April–May	[35,36]
	Dog, New Guinea Singing	*Canis hallstromi*	Papua New Guinea	Yes	August–October	[18,65]
	Ethiopian wolf	*Canis Simensis*	Ethiopia	Yes	August–November	[44,66]
	Golden jackal	*Canis aureus*	Europe, Africa, Middle East, Central and Southeast Asia	Yes	Varies	[67]
	Grey wolf	*Canis lupus*	North America, Europe and North and Central Asia	Yes	Late January– March	[43]
	Red wolf	*Canis rufus*	USA	Yes	January–March	[29]
	Side striped jackal	*Canis adustus*	Africa	Yes	June–July	[63]
South American foxes	Chilla	*Pseudalopex griseus*	South America	Yes	August–September	[68]
	Crab eating fox	*Cerdocyon thous*	South America	Yes	June–September	[11]
	Culpeo	*Pseudalopex culpaeus*	South America	Yes	Male: June–mid-OctoberFemale, August–October	[69]
	Hoary fox	*Lycalopex vetulus*	South America	Yes	July–September	[70]
Other	Bat eared fox	*Otocyon megalotis*	Africa	Yes	June–July	[71]
	Bush dog	*Speothos venaticus*	South America	No	-	[15]
	Bengal fox	*Vulpes bengalensis*	South Asia	Yes	December–January	[72]
	Grey fox	*Urocyon cinereoargentues*	Central and North America	Yes	January–April	[73]
	Island fox	*Urocyon littoralis*	United States	Yes	February–mid March	[19]
	Maned wolf	*Chrysocyon brachyurus*	South America	Yes	Southern hemisphere, March–May, North America, October–January	[32,37]
	Raccoon dog	*Nyctereutes procyonoides*	Northern and Eastern Europe	Yes	February–March	[40,49]

**Table 2:** Sperm collection methods in wild canid results (Avg ± Standard error of mean). n.r. = not reported.

Technique	Species	N Animals (samples)	Total Sperm (×10^6^ cells)	Motility (%)
Electroejaculation	African wild dog [107]	7 (7)	~127.4 ± 52.7	69.5 ± 3.3
	African wild dog [108]	17 (17)	30.5 ± 9.7	55.0 ± 6.3
	African wild dog [51]	20	32.3 ± 9.2	47.4 ± 6.7
	Coyote [109]	15	~63 ± 12.6	~90
	Coyote [30] (peak season/January)	10	917.2 ± 497.2	90.4 ± 4.5
	Maned wolf [110]	14 (25)	78.1 ± 35.0	59.8 ± 4.9
	Mexican grey wolf [111]	4 (27)	756.2 ± 153.9	~90% ^†^
	Grey wolf [111]	7 (13)	1597.4 ± 390.4	n.r.
	Grey wolf [95]	7 (7)	n.r.	~70
	Red wolf [112]	15 (31)	470.0 ± 83.5	69.6 ± 3.5
	Red wolf [113]	15 (37)	349.4 ± 51.1	75.6 ± 2.6
	Red wolf [99]	39 (38)	720.0 ± 287.5	80.8 ± 16.9
Epididymal Collection	Dingo dog [114]	12	873.0 ± 229	n.r.
	Grey wolf [115]	9–13	69.3 ± 23.3 *	n.r.
Manual/Digital Manipulation	Arctic fox [42] (N:6L:N photoperiod)	4 (11)	~ 228.8 ± 25.2	86.0 ± 2.0
	Crab-eating fox [103]	2 (13)	217.4 ± 84.3	68.0 ± 6.1
	Maned wolf [104]	3 (70)	73.9 ± 10.4	76.1 ± 2.9
	Raccoon dog [102]	20 (20)	~0.06	57.2
	Red (silver) fox [116]	17 (45)	~79.8 ± 5.6	70.3 ± 2.5 ^†^
Urethral catheterization	African wild dog [100]	1 (1)	216.0	93.0
	Maned wolf [100]	1 (1)	1.0	40.0
	Maned wolf (unpublished data)	2 (2)	1.4 ± 1.3	80.0 ± 20.0
	Red wolf [99]	8 (8)	30.1 ± 35.1	36.3 ± 13.2

* Data does not include aggregated sperm. ^†^ Numbers reflect progressive/forward motile sperm.

**Table 3:** Epididymal sperm collection during breeding (grey shading) versus non-breeding (white) seasons (*unpublished data*). dnc = did not count.

Species	ID	Age	Month	Spermic	Total Sperm (×10^6^ cells)	Motility (%)
African Wild Dog	116047 ^#^	7 y	September-18	Yes	88.0	65
	6217	-	January-19	Yes	dnc	0
	5639 *	1 y	June-20	Yes	2.7	dnc
	5640 *	1 y	June-20	Yes	[Too low]	50
	5641 *	1 y	June-20	Yes	17.6	55
	5642 *	1 y	June-20	No	-	-
	5643 *	1 y	June-20	Yes	2.4	10
Maned Wolf	3382	5 y	June-18	No	-	-
	3206	8 y	December-18	Yes	46.0	70
	3230 ^†#^	8 y	August-19	Yes	23.5	60–65
	2954	12 y	October-19	No	-	-
	3153	10 y	June-20	Yes	[Too low]	Few
	3176	10 y	December-20	Yes	4.8	50
Red Wolf	1460	12 y	July-17	No	-	-

* Denote littermates. ^†^ Indicates sperm only collected on a single epididymis. ^#^ Indicates collections taking place >24 h following animal death/necropsy.

## Data Availability

The data presented in this study are available on request from the corresponding author. The data are not publicly available as studies are in preliminary stage.
